# Optimizing LED photobiomodulation parameters to prevent cartilage matrix degradation in knee osteoarthritis: in vitro and in vivo study

**DOI:** 10.1186/s13018-025-06341-7

**Published:** 2025-10-29

**Authors:** Tianxiang Fan, Peng Xia, Safayet Ahmed, Yuen H. Tsang, Ye Li, Siu Ngor Fu

**Affiliations:** 1 Department of Rehabilitation Sciences, The Hong Kong Polytechnic University, Hong Kong, China; 2 Department of Physics, Oregon State University, Corvallis, Oregon USA; 3 Department of Applied Physicals, The Hong Kong Polytechnic University, Hong Kong, China

**Keywords:** Osteoarthritis, Photobiomodulation, Light-emitting diode, Cartilage, Knee pain

## Abstract

**Background:**

Photobiomodulation using light-emitting diode (LED) therapy is a promising and safe non-invasive approach for treating knee osteoarthritis (KOA), yet the most effective wavelength and energy density to prevent cartilage matrix degradation remain unclear. This study aimed to determine the optimal LED parameters for suppressing chondrocyte extracellular matrix (ECM) degradation in vitro, and for alleviating ECM degradation and pain-related behaviours in a murine KOA model.

**Methods:**

TNF-α-stimulated RCJ 3.1.C5.18 chondrocytes were irradiated with LEDs (625/810/940/1050 nm; 13–78 J/cm^2^ at 44 mW/cm^2^). Chondrocyte ECM-related markers (*mmp3, mmp13, col2a1, aggrecan*) were quantified via RT-qPCR. In vivo, destabilization of the medial meniscus (DMM)-induced KOA mice (n = 5–6/group) received 810 nm (39/52 J/cm^2^) or 940 nm (39/52 J/cm^2^) LED therapy thrice weekly for 6 weeks. Pain-like behaviors (Von Frey, incapacitance tests) and histopathology (OARSI scoring, mmp3/collagen II immunofluorescence) were assessed.

**Results:**

In vitro, 940 nm LED at 52 J/cm^2^ most effectively suppressed *mmp3/mmp13* mRNA (P < 0.05) and upregulated *col2a1/aggrecan* (*P* < 0.05), while 810 nm at 39 J/cm^2^ only inhibited *mmp3* and *mmp13*. The LED with 625 nm and 1050 nm showed no significant effect on chondrocyte ECM degradation markers. In vivo, 940 nm at 52 J/cm^2^ improved weight-bearing asymmetry by 31% at 6 weeks (vs. DMM controls, *P* = 0.03) and reduced cartilage degradation by 50% (OARSI score, *P* = 0.03). Immunofluorescence indicated that 940 nm at 52 J/cm^2^ LED therapy significantly inhibited the expression of mmp3 and increased collagen II protein levels in cartilage tissues.

**Conclusion:**

LED therapy at 940 nm with 52 J/cm^2^ attenuates ECM degradation and pain in KOA, defining a target LED parameter set for potential clinical translation.

**Supplementary Information:**

The online version contains supplementary material available at 10.1186/s13018-025-06341-7.

## Introduction

Osteoarthritis (OA) is one of the most common joint disorders characterized by progressive articular cartilage degeneration, synovitis and osteophyte formation, leading to chronic joint and functional disability [1, 2]. Central to OA pathogenesis is cartilage extracellular matrix (ECM) degradation, driven by an imbalance in chondrocyte catabolic-anabolic homeostasis [3]. Matrix metalloproteinases (MMPs) play important roles in cartilage destruction by cleaving key ECM components such as collagen type II and aggrecan [4]. Despite extensive research, no disease-modifying osteoarthritis drugs (DMOADs) have been clinically approved, underscoring the urgent need for novel therapeutic strategies targeting ECM degradation [5]. Recent efforts have shown the potential of orthobiological approaches in protecting cartilage, such as mesenchymal stem cells, Wharton's jelly, bone marrow aspirate concentrate, although their clinical efficacy remains to be further validated [6–8].

Alongside these biological strategies, non-invasive modalities such as photobiomodulation (PBM) have been introduced for OA management, utilizing light-emitting diode (LED) or laser irradiation to modulate cellular processes, promote tissue repair, and alleviate pain [9–13]. Compared to laser-based PBM, LED therapy offers distinct advantages, including broader tissue coverage, reduced cost, and enhanced safety profiles, making it particularly suitable for joint applications [14–16]. Critically, the therapeutic efficacy of LED therapy is governed by two key parameters: wavelength, which influences the biological response by targeting specific chromophores, and energy density, which exhibits a biphasic dose–response and must fall within an effective therapeutic window [17, 18].

While previous studies have explored the effects of red to near-infrared (NIR) light (600–900 nm) in OA models, limitations remain [19, 20]. First, existing studies have not directly compared the efficacy of distinct wavelengths and energy density in regulating chondrocyte ECM metabolism. Second, the biological effects of NIR wavelengths beyond 1000 nm on cartilage homeostasis remain unexplored [19, 21–23]. Third, while red and infrared LEDs show partial suppression of chondrocyte degradation in vitro [24, 25], the energy density thresholds required for robust ECM preservation remain poorly defined [18]. To address these limitations, we selected four representative wavelengths (625, 810, 940, and 1050 nm) covering the red to NIR spectrum, based on previous studies on musculoskeletal disorders and pain [26]. We also tested energy densities ranging from 13 to 78 J/cm^2^, reflecting the commonly recognized therapeutic window in PBM applications [26].

Given the heterogeneity of OA, with varying pathological mechanisms across endotypes, such as cartilage degradation-driven endotypes, tailoring LED parameters to specific OA subtypes is essential for achieving targeted therapeutic effects. Previous attempts to apply LED therapy in advanced OA stages have yielded limited success. In rabbit models of late-stage knee OA (KOA), LED interventions increased type II collagen expression but failed to restore aggrecan or suppress *mmp3/mmp13* activity, indicating incomplete ECM restoration [27, 28]. Furthermore, gait analysis in rodent late-stage OA models revealed no significant improvement in pain-related behaviors following LED treatment [27]. These findings collectively suggest that late-stage OA may represent a phase in which structural damage surpasses the reparative capacity of LED therapy. In contrast, emerging evidence posits early-stage OA as a critical “therapeutic window” wherein targeted interventions could halt cartilage catabolism and reestablish joint homeostasis [29]. The surgical destabilization of the medial meniscus (DMM) model provides an ideal platform for studying early intervention strategies. This well-validated murine model recapitulates human OA progression, with histopathological analysis confirming measurable cartilage thinning within one week post-surgery—a hallmark of incipient OA [30, 31].

Therefore, this study aimed to address these gaps by (1) comparing the effects of LEDs with four wavelengths (625 nm, 810 nm, 940 nm, 1050 nm) on chondrocytes, (2) identifying the optimal energy density for chondrocyte treatment, and (3) evaluating the effective LED parameters in early-stage of knee OA mouse induced by DMM surgery.

## Materials and methods

### Cell culture

To investigate the effectiveness and optimal wavelength and energy density of LED on ECM degradation, RCJ C5.18 cells (rat chondrocyte cell line) were used. Cells were maintained in DMEM/F12 (11,320,033, Thermo Fisher, USA) medium supplemented with 10% fetal bovine serum (Gibco, Brazil, 10,270,106) and 100 U/mL penicillin–streptomycin (HY-K1006, MedChemExpress, USA) in an incubator at 37 °C with 5% CO_2_. To simulate chondrocyte ECM degradation, C5.18 chondrocyte cells were seeded at 1 × 10^5^ cells/well in 12-well plates and then treated with recombinant rat TNF-α (P6335, Beyotime, China) at concentrations of 20 ng/mL for 24 h prior to LED irradiation, with an unstimulated control group [32].

### Light-emitting diode bio-stimulation

**Fig. 1:**
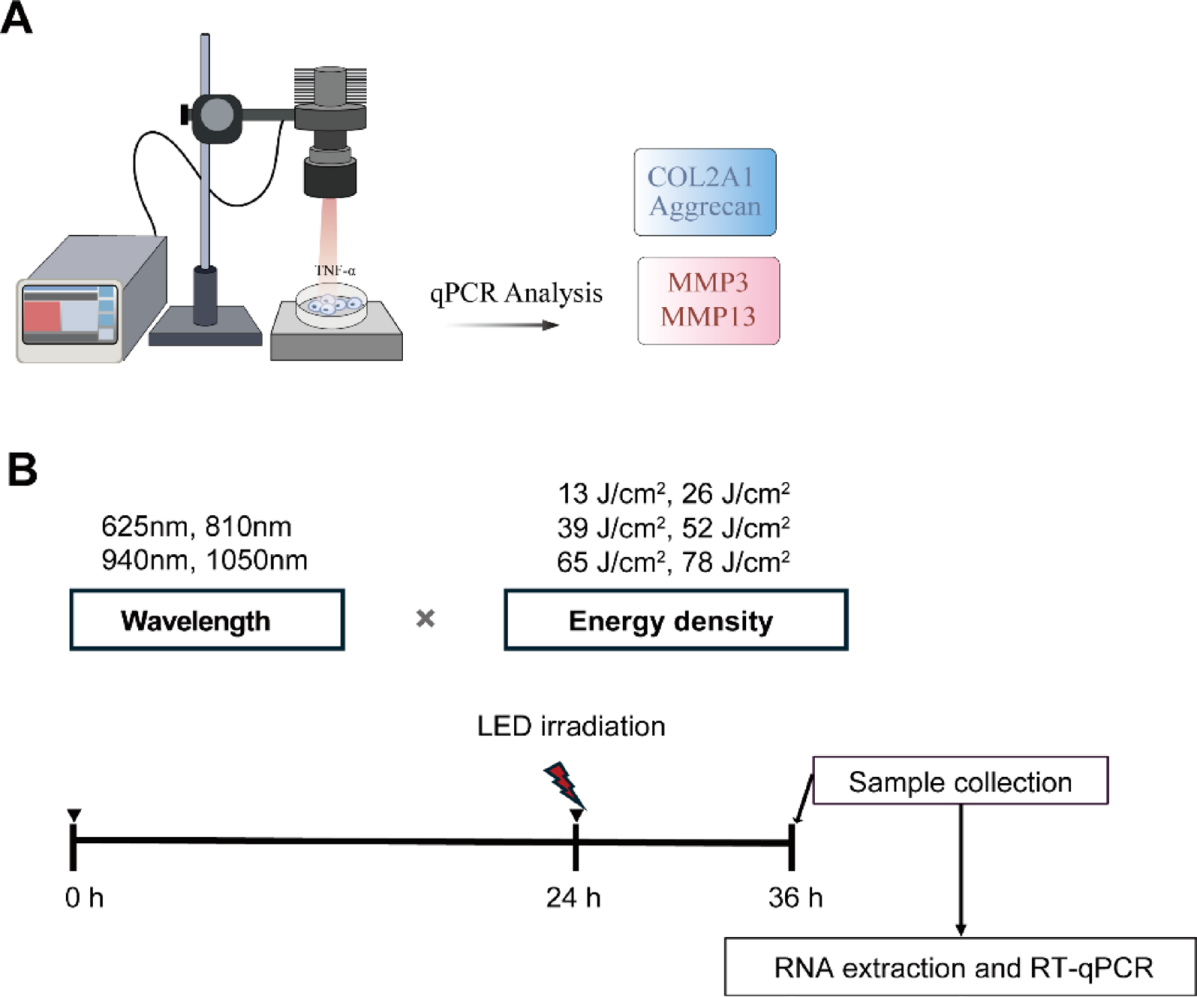
LED irradiation process for cell study. **A** LED irradiation of cells at varying wavelengths and energy densities, followed by RT-qPCR analysis. **B** schematic representation of cell experimental procedure

To measure power density and irradiate cells, the LED light (single LED) was connected to the adjustable collimation optic (SM1U25-B, ThorLabs, USA) and was secured on the stand (Fig. 1). The adjustable collimation optic was adjusted to ensure the illuminating area covered exactly one well of the 12-well plate. The power energy meter (PM100D, ThorLabs, USA) was placed below the LED light source at a distance of 12 cm, and the channel LED driver (DC2200, ThorLabs, USA) was adjusted to achieve a power density of 44 mW/cm^2^. Then, the transparent 12-well plate was placed under the LED light source, and the area between the LED light source and the bottom of the plate was adjusted to be 12 cm. The cells were treated with LED of 625 nm (M625L4, THORLABS, USA), 810 nm (M810L5, THORLABS, USA), 940 nm (M940L3, THORLABS, USA), 1050 nm (M1050L2, THORLABS, USA) or remained unstimulated. The time of radiation to cells was 5 min, 10 min, 15 min, 20 min, 25 min, 30 min to achieve energy density of 13 J/cm^2^, 26 J/cm^2^, 39 J/cm^2^, 52 J/cm^2^, 65 J/cm^2^, and 78 J/cm^2^ respectively. All samples, including both irradiated and control groups, were kept at room temperature for a total of 30 min to ensure consistent exposure conditions. For the control groups (non-irradiated cells), the culture plates were covered with aluminum foil during the entire 30-min period. Detailed light source specifications and parameters are provided in Supplementary Table 2. Cells were harvested after 12 h of LED irradiation. Subsequently, total RNA was extracted from the cells for RT-qPCR analysis.

The energy density applied during cell irradiation is challenging to determine accurately when transitioning to animal studies, as part of the light is absorbed by the skin, other tissues, and the cell medium. Nevertheless, one study empirically demonstrated that direct application of cell-optimized PBM parameters achieved conserved chondroprotective efficacy in vivo, mirroring in vitro outcomes [24]. Leveraging this translational evidence, we maintained identical PBM parameters (810/940 nm; 39/52 J/cm^2^) across experimental platforms to confirm conserved anti-catabolic effects. After shaving the skin cleanly around the mouse knee joint using a shaver, the mice were then placed into a restrainer where their position was gently secured. The LED light was applied specifically to the anterior surface of the knee joint. LED irradiation was delivered at 810 nm or 940 nm with an energy density of 52 J/cm^2^ for 20 min, or 39 J/cm^2^ for 15 min. The mice in the sham-irradiated controls were also restrained for the same duration but did not receive LED therapy.

### RNA extraction and quantitative RT-PCR

Total RNA was extracted using RNAiso Plus (9109, Takara Bio, Japan) and cDNA was synthesized using PrimeScript™ RT Master Mix (Perfect Real Time) (RR036A, Takara Bio, Japan) according to the manufacturer’s instructions. The reverse reactions were incubated at 37 °C for 15 min followed by inactivation at 85 °C for 5 s. To quantitatively analyze RNA transcript levels, RT-qPCR was performed using TB Green® Premix Ex Taq™ (RR420A, Takara Bio, Japan) on CFX Connect Real-Time PCR Detection System (12,011,319, Bio-Rad Ltd, USA). Cycles of 95 °C for 30 s, followed by 39 cycles of 95 °C for 5 s and 60 °C for 30 s, were employed as the cycling conditions. The primers used were showed in supplementary Table 1. mRNAs levels of GAPDH were used as internal references.

### Animal study design

Male C57BL/6 mice, 12 weeks of age, were used in this study, with adherence to animal ethics approval (22–23/425-RS-R-OTHERS) from The Animal Subjects Ethics Sub-committee of The Hong Kong Polytechnic University). Mice were randomly assigned to six groups by computer-generated randomized code (n = 6 per group). During surgery, five mice died due to anesthesia-related complications and were excluded from analyses, resulting in a final group size of 5–6 per group. Baseline measurements of withdrawal threshold were taken before the surgeries. One week post-surgery, assessments of weight-bearing asymmetry were conducted before the first LED irradiation session. The LED irradiation treatments, employing wavelengths of either 940 nm, 810 nm, or sham irradiation, were administered three times per week for a total duration of six weeks (Fig. 4). Eight weeks after the surgery, evaluations of withdrawal threshold, and weight-bearing asymmetry were performed before euthanasia. The knees were then harvested for histological analysis to assess the extent of cartilage damage.

### Establishment of KOA animal model

Mice were anesthetized via intraperitoneal injections of Ketamine (100 mg/kg) and Xylazine (2 mg/kg). To establish the DMM-induced OA model, hair around the right knee was shaved and the skin disinfected. A small medial parapatellar incision was made under a microscope, and the medial meniscotibial ligament was carefully transected to destabilize the medial meniscus, as previously described [reference] [33, 34]. For sham operations, the medial meniscotibial ligament was identified but left intact; Finally, the joint capsule and skin were sutured, and the area was disinfected with povidone-iodine. Following surgery, mice were placed in clean cages with warm water bags surrounding them for warmth, ensuring their comfort and facilitating recovery. In terms of postoperative care, Buprenorphine (Temgesic, 0.3 mg/mL) was administered subcutaneously twice a day for three days, and Meloxicam oral suspension (1.5 mg/mL) was added to the drinking water of the mice for three days for analgesia. Furthermore, Enrofloxacin (200 mg/L in water, 2.5%) was added to the drinking water of the mice for seven days to prevent postoperative infection, ensuring a thorough and humane approach to animal care throughout the study.

### Incapacitance test for spontaneous pain

To assess the differences in hind paw weight distribution between the right and left (control) rear limbs, a mouse incapacitance tester was used (BIO-SWB-TOUCH-M, Bioseb, France). Briefly, mice were first acclimatized to the chamber one day prior to the first experimental measurements. Mice were placed on a separate force plate in a test chamber with their hind paws and maintained in position by lightly holding the tail. Care was taken to ensure that the body did not touch the wall of the incapacitance chamber during recording. The body weight distribution between the hind paws was measured over a period of 5 sec [35]. The incapacitance test was performed three consecutively times without intervals, and the data are presented as the mean of the three tests. Data are expressed as the percentage of body weight (g) distributed on the right limb according to the following equation: % = weight on right limb (ipsilateral) / weight on left limb (contralateral) × 100%.

### Electronic Von Frey test for evoked pain

To assess evoked pain-like behaviors, mice were first acclimated to individual acrylic cages (12 × 30 × 31 cm per cage) for 30 min in a quiet room, one day prior to the experimental measurements. On the day of testing, mice were acclimated for 30 min in individual chambers on top of a wire grid platform until exploratory behaviors ceased [36]. Mechanical sensitivity was measured by determining the hind paw-withdrawal threshold with electronic Von Frey (38450, Ugo Basile Inc., Italy). The electronic Von Frey meter consisted of a force transducer fitted with a rigid stimulator filament in nitinol. During the procedure, the filament was applied perpendicularly to the hind paw with increasing force until paw withdrawal, licks, or shakes of the paw occurs, which considered as positive response [37]. Upon detection of a positive response, the instrument automatically recorded the peak applied force when the observer steps on the pedal. Each mouse underwent the test three times, with a 10-min inter-stimulus interval between each measurement. To obtain the final threshold value, the average of the three hind paw withdrawal threshold forces (in grams) was calculated [38].

### Histological analysis and Immunofluorescence analysis

After euthanizing the mice, knee joints were fixed in 4% paraformaldehyde for 48 h after being dissected free of skin and excess muscle. Perfusion was not performed. Samples were decalcified in 0.5 M EDTA for 7 days, on a shaker at 37 °C (70 rpm), with the decalcification solution replaced every two days [39]. The samples were then meticulously rinsed, subjected to a graded alcohol dehydration process, infiltrated with paraffin, and ultimately embedded in paraffin in a frontal orientation. The paraffin-embedded blocks were sectioned with 4 μm thickness. These sections were then deparaffinized in xylene, gradually hydrated through graded ethanol solutions, and subsequently stained utilizing Safranin O and Fast Green. The Osteoarthritis Research Society International (OARSI) ECM degradation scores were assessed on 13 serial levels collected at approximately 80 μm intervals across each knee joint [40]. The highest OARSI grade was recorded for each section across the tibiofemoral surfaces, and the joint-level score was defined as the maximum value among the 13 sections. Three independent individuals completed this grading in a blinded manner. The graders were unaware of the experimental group of each sections. Although the person performing the staining knew the group assignments, slices selection was conducted by another individual to minimize potential bias.

The tissue sections were dewaxed and prepared for immunofluorescence staining, followed by antigen retrieval using Citrate Antigen Retrieval Solution (C1032-100 ml, Solarbio, China), with heating in a 62 °C water bath for 12 h. The membranes were permeabilized with 0.5% Triton (ST797-100 ml, Beyotime, China) for 30 min, then blocked with 5% BSA (B824162-10 g, Macklin, China) for one hour. Subsequently, the sections were incubated overnight at 4 °C with primary antibodies (diluted 1:100 in BSA; mmp3: Abcam Cat# ab52915; Collagen II: Cat# ab34712). After three washes with PBS, the sections were incubated with Alexa Fluor 488-conjugated secondary antibodies for one hour at 37 °C. DAPI (F6057-20ML, Sigma-Aldrich, USA) was used to stain the nuclei.

Fluorescence images were captured using a Nikon Ti2-E microscope and analyzed using ImageJ software. Quantification of signal intensity of Collagen II in regions of interest (ROI) was performed using the mean fluorescence intensity (MFI) method, where the final MFI was calculated as: Final MFI = MFI of ROI – MFI of background, as previously described [41]. For immunofluorescence analysis, we retained the sections that were positioned between the 80 μm-spaced histology slices. From each joint, three of these intervening sections were randomly selected, and the mean MFI value of these sections was used as the representative score for that sample, and the average MFI from these three slices was used as the representative score for the sample. The number of mmp3-positive cells within the ROI was calculated and divided by the total cell count in the same area.

### Statistical analysis

Data were expressed as mean ± standard deviation (SD). Statistical analyses were completed with Prism GraphPad (version 9.1.1). Unpaired Student’s t-tests were used to compare differences between two groups for qPCR tests. For data that did not follow a normal distribution, Mann–Whitney test was used. Kruskal–Wallis rank sum test was used to analyze the difference between groups in von Frey test and incapacitance test. The Mann–Whitney test was used to evaluate the differences in OARSI scores between groups. Sample size was based on a previous power calculation using OARSI scores in DMM mice (mean difference = 0.5, SD = 0.2), which indicated that n = 3 per group would be sufficient to detect a significant difference with 80% power and an alpha level of 0.05 [42]. To ensure robustness and account for potential experimental variability, 6 mice per group were included. A two-sided *P* < 0.05 was considered statistically significant. Analyses were performed using R software version 4.1.3 or GraphPad version 9.5.0. All reported *P* values are calculated from two-sided comparisons. OpenAI’s GPT-4o was used solely for grammar checking to ensure grammatical accuracy in the manuscript. No content generation or data analysis was performed using AI.

## Results

### Effects of LED therapy on mRNA expressions of extracellular matrix degradation in chondrocyte cell line C.518

**Fig. 2:**
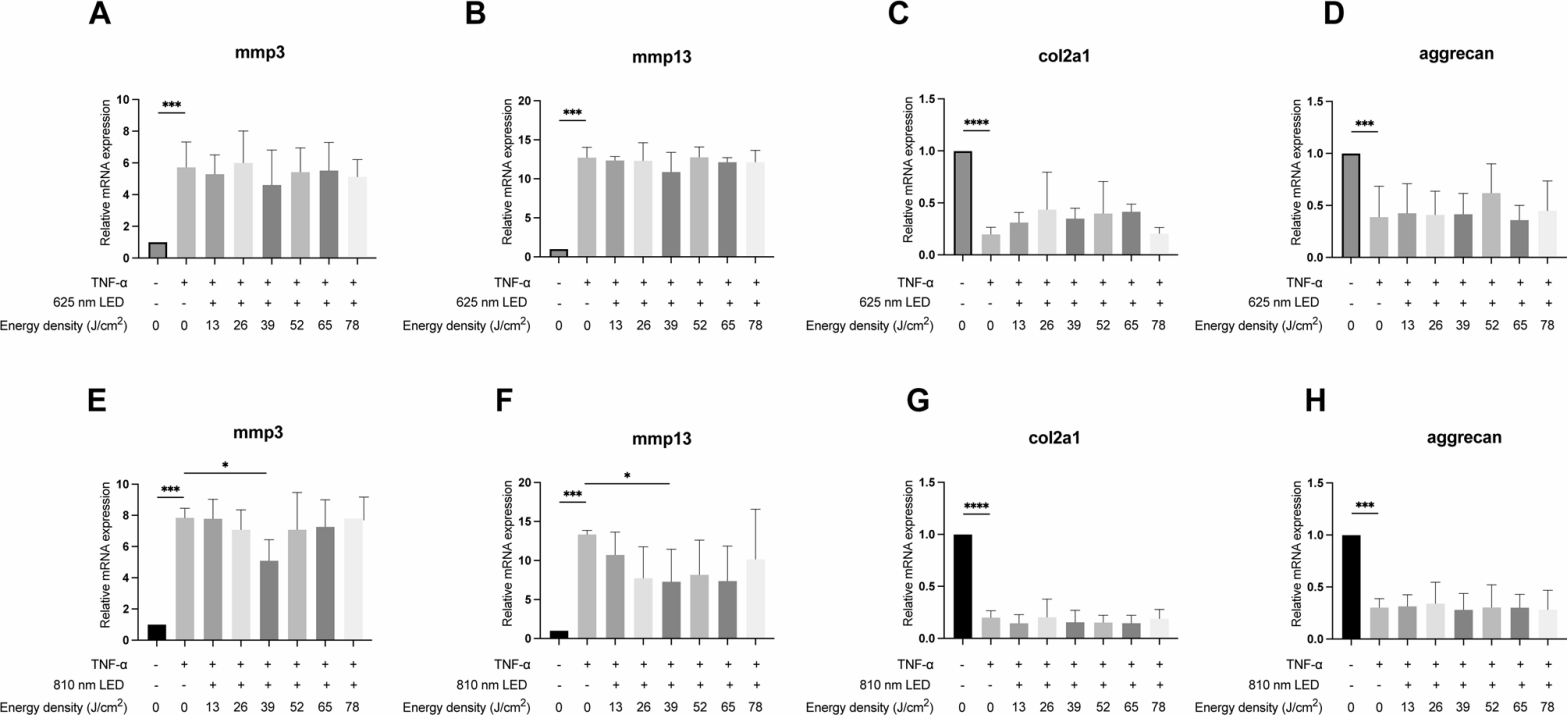
Effects of 625 nm and 810 nm LED irradiation on TNF-α stimulated chondrocyte cell line C.518. (**A**–**D)** After recombinant TNF-α stimulation (20 ng/mL), RT-qPCR analysis of *mmp3* (**A**), *mmp13* (**B**), *col2a1* (**C**), and *aggrecan* (**D**) in chondrocyte cell line C.518 following irradiation with different energy densities of 625 nm LED; (E–H) After recombinant TNF-α stimulation (20 ng/mL), RT-qPCR analysis of *mmp3* (**E**), *mmp13* (**F**), *col2a1* (**G**), and *aggrecan* (**H**) in chondrocyte cell line C.518 following irradiation with different energy densities of 810 nm LED. Student’s t-test or Mann–Whitney test was used for comparison. n = 3 for each group. *P < 0.05, **P < 0.01, ***P < 0.001, ****P < 0.0001

To investigate the effectiveness and optimal wavelength and energy density of LED on ECM degradation, rat chondrocyte cell line C5.18 was used. The results showed a significant increase in the mRNA levels of *mmp3* and *mmp13*, and a significant suppression of *col2a1* and *aggrecan* following TNF-α stimulation. Using 625 nm LED at energy densities ranging from 13 to 78 J/cm^2^ did not significantly inhibit chondrocyte degeneration (Fig. 2A–D). However, 810 nm LED at 39 J/cm^2^ significantly inhibited the mRNA levels of *mmp3* and *mmp13* (Fig. 2E, F).

**Fig. 3:**
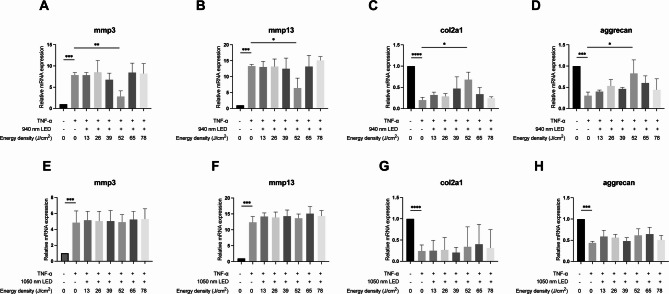
Effects of 940 nm and 1050 nm LED irradiation on TNF-α stimulated chondrocyte cell line C.518. (**A**–**D**) After recombinant TNF-α stimulation (20 ng/mL), RT-qPCR analysis of *mmp3* (**A**), *mmp13* (**B**), *col2a1* (**C**), and *aggrecan* (**D**) in chondrocyte cell line C.518 following irradiation with different energy densities of 940 nm LED; (**E**–**H**) After recombinant TNF-α stimulation (20 ng/mL), RT-qPCR analysis of *mmp3* (**E**), *mmp13* (**F**), *col2a1* (**G**), and *aggrecan* (H) in chondrocyte cell line C.518 following irradiation with different energy densities of 1050 nm LED; Student’s t-test or Mann–Whitney test was used for comparison. n = 3 for each group. *P < 0.05, **P < 0.01, ***P < 0.001, ****P < 0.0001

To further investigate whether longer wavelengths have different effects on inhibiting chondrocyte degeneration, experiments under the same conditions using LED light at 940 nm and 1050 nm with chondrocyte cell lines were conducted. The results showed that 52 J/cm^2^ of 940 nm LED significantly suppressed the levels of *mmp3* and *mmp13*, while significantly increasing the mRNA levels of *col2a1* and *aggrecan* (Fig. 3A–D). In contrast, the 1050 nm LED, at various energy densities, did not demonstrate a significant inhibitory effect on chondrocyte ECM degradation (Fig. 3E–H).

### Effects of LED therapy on mice with knee osteoarthritis for pain-like behaviors

**Fig. 4:**
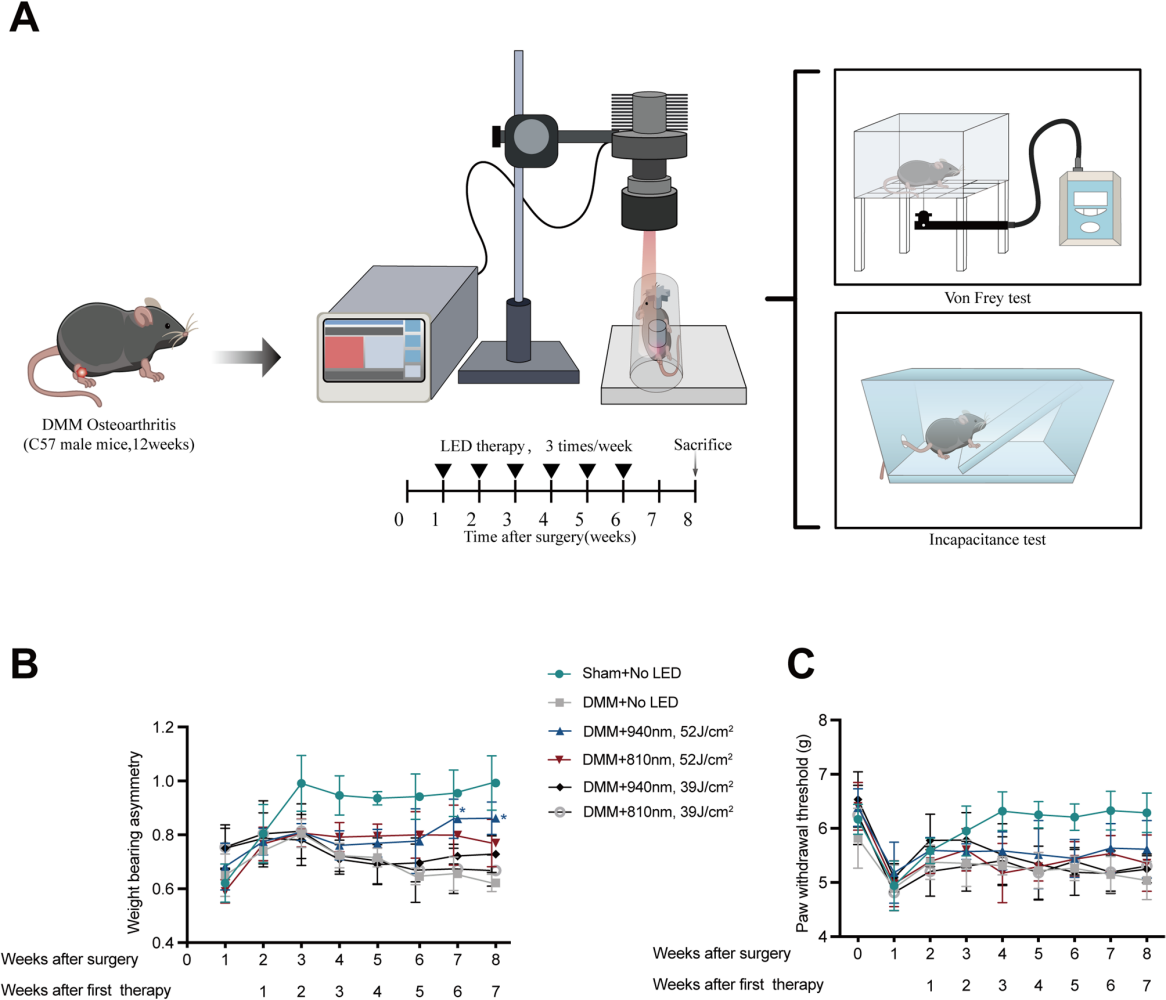
LED therapy at 940 nm with 52 J/cm^2^ improved weight-bearing asymmetry in mice with knee osteoarthritis. **A** Schematic representation of animal experimental procedure, **B** knee incapacitance tests were evaluated once a week. Data (average of the three hind paw withdrawal threshold forces (in grams)) are presented as means ± SD, **C** Paw withdraw thresholds were tested by electricity Von Frey filament once a week. Data (average of the three hind paw withdrawal threshold forces (in grams)) are presented as means ± SD; n = 5 in sham + no LED, DMM + no LED, DMM + 940 nm at 52 J/cm^2^, DMM + 810 nm at 52 J/cm^2^ and DMM + 810 nm at 39 J/cm^2^. n = 6 in DMM + 940 nm at 39 J/cm^2^; Statistical analyses were conducted by Kruskal–Wallis rank sum test. *P < 0.05; DMM: surgical destabilization of the medial meniscus; LED: Light-emitting dioxide

We performed incapacitance tests to assess the differences in the weight-bearing distribution between operated and non-operated hind paws (Fig. 4B). Three weeks post-operatively, the sham-operated group exhibited a marked increase in weight distribution on the ipsilateral hind paw compared to the DMM groups, which then remained consistent in the subsequent weeks. LED therapy at 940 nm/52 J/cm^2^ improved ipsilateral weight-bearing asymmetry by 31% compared with untreated DMM mice at week 6 since first therapy (*P* = 0.03) (Fig. 4B). Regarding the DMM group without LED treatment, an increase in weight distribution on the ipsilateral hind paw was noted from the first week to the third week post-surgery, following which it declined and stabilized from the fifth week onwards. In the case of the 810 nm LED with 39 J/cm^2^, 810 nm with 52 J/cm^2^ and 940 nm with 39 J/cm^2^ group, an increase in weight distribution on the ipsilateral side was increased from the first week of therapy, maintaining stability until the study concluded. These findings suggested that 940 nm LED with 52 J/cm^2^ can reduce spontaneous pain-like behavior in mice with KOA.

Paw withdrawal threshold was measured using the electricity Von Frey test on the ipsilateral paws (Fig. 4C). One week post-surgery, a decrease in the withdrawal thresholds was noted across all groups. Four weeks after surgery, the group with DMM but without LED therapy exhibited a significantly higher withdrawal threshold compared to the other three groups. Nevertheless, by the end of the monitoring period, there were no substantial differences in withdrawal thresholds between the DMM without LED group and the groups treated with either 940 nm or 810 nm LED. Following six weeks of therapy with 940 nm LED with 52 J/cm^2^, there was a trend towards alleviation of evoked pain, though the difference remained statistically insignificant when compared with the DMM without LED group.

### Effects of LED therapy on mice with knee osteoarthritis for cartilage degradation

**Fig. 5:**
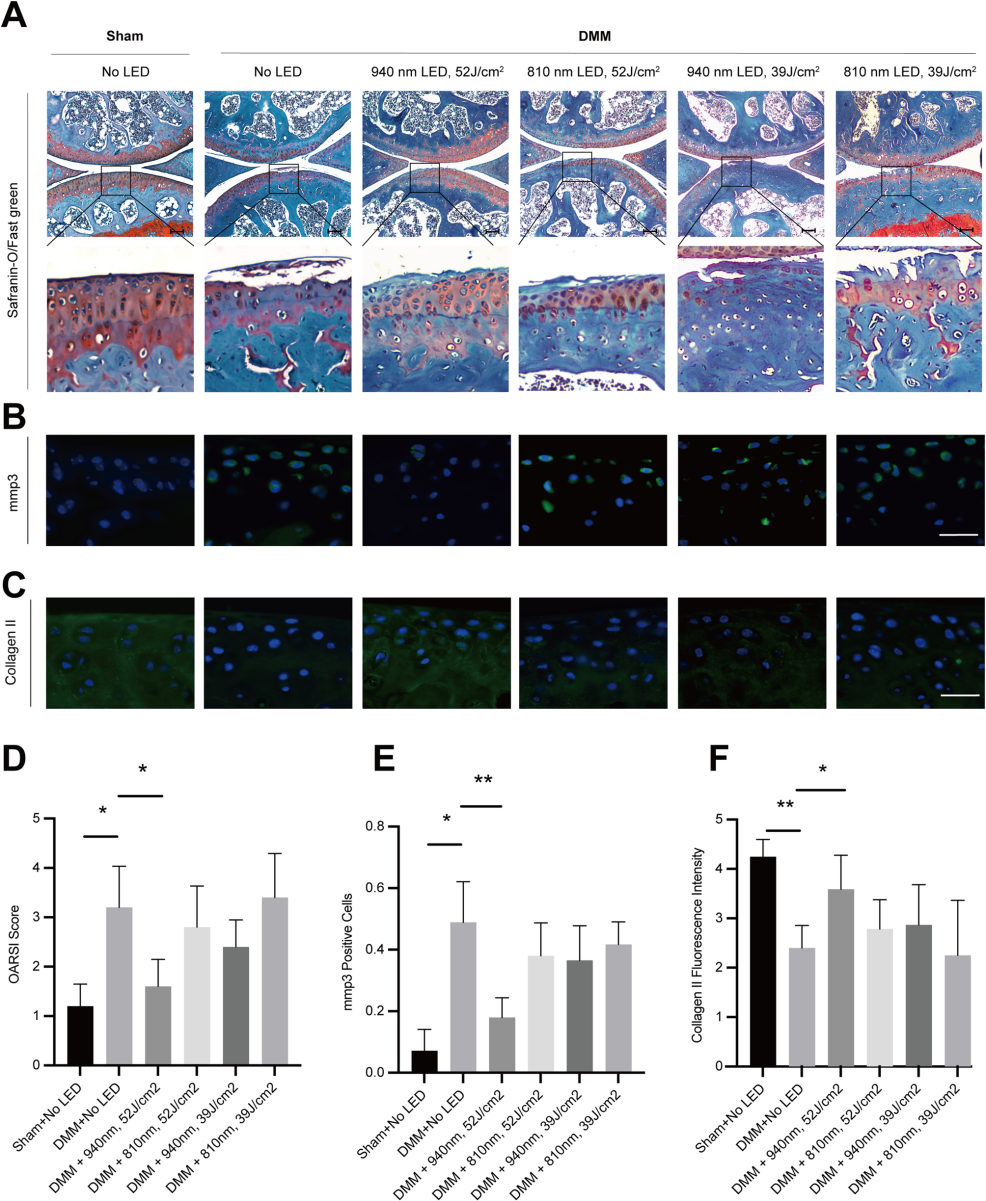
LED therapy at 940 nm with 52 J/cm^2^ inhibited ECM degradation in mice with knee osteoarthritis. **A** Safranin-O/Fast green (SOFG) staining was performed to assess the degree of knee cartilage degeneration. Scale bars, 100 μm; **B**, **C** immunofluorescence detected the expression of mmp3 and Collagen II in articular cartilage. Nuclei are stained blue (DAPI) and MMP3 or Collagen II are stained green. Scale bars, 10 μm; **D** osteoarthritis Research Society International (OARSI) Osteoarthritis Cartilage Histopathology Assessment System was used to evaluate the articular cartilage degradation; **E** quantification of mmp3 Immunofluorescence positive cells; **F** quantification of Collagen II. n = 5 in sham + no LED, DMM + no LED, DMM + 940 nm at 52 J/cm^2^, DMM + 810 nm at 52 J/cm^2^ and DMM + 810 nm at 39 J/cm^2^. n = 6 in DMM + 940 nm at 39 J/cm.^2^; The Mann–Whitney test was used for comparison between two groups, *P < 0.05, **P < 0.01

To compare the effectiveness of 810 nm and 940 nm LEDs, both applied at the same energy density, on ECM degradation in DMM-induced KOA mice, we used SO-FG staining and OARSI scoring for cartilage damage evaluation. The DMM group showed a statistically significant increase in ECM degradation relative to the sham group. When comparing DMM mice treated with LEDs to those without LED treatment, only the 940 nm LED with 52 J/cm^2^ treatment resulted in a significantly 50% lower cartilage damage score (*P* = 0.03), whereas the 940 nm with 39 J/cm^2^, 810 nm with 39 J/cm^2^ and, 810 nm with 52 J/cm^2^ did not show significant protective effects on cartilage (Fig. 5A, D). These findings suggest that 940 nm LED therapy with 52 J/cm^2^ effectively inhibits ECM degradation in KOA mice. To further investigate the impact of LED treatment on cartilage anabolic and catabolic factors, immunofluorescence analysis was performed. The 940 nm LED at 52 J/cm^2^ significantly reduced the protein levels of mmp3 and increased collagen II in the cartilage tissues (Fig. 5B–F).

## Discussion

This study aimed to determine the effective parameters of LED therapy for cartilage matrix degradation through both in vitro and in vivo studies. The results demonstrated that LED therapy at 940 nm and 52 J/cm^2^ was more effective in promoting cartilage synthesis alongside inhibiting degradation than 625 nm, 810 nm and 1050 nm LED from 13 to 78 J/cm^2^. In DMM-induced KOA mice, 940 nm LED at 52 J/cm^2^ improved spontaneous pain, but had no significant effect on evoked pain. The 810 nm LED at both 39 J/cm^2^ and 52 J/cm^2^, as well as the 940 nm LED at 39 J/cm^2^, had no effect on either spontaneous or evoked pain. Histological analysis further revealed that only the 940 nm LED at 52 J/cm^2^ inhibited the expression of mmp3 in cartilage tissue, increased collagen II levels, and effectively inhibited cartilage degeneration. This study highlights that 940 nm LED at 52 J/cm^2^ is the most effective parameter among those tested for improving cartilage metabolism, reducing degeneration, and alleviating spontaneous pain in early-stage KOA.

Some previous studies investigated the effects of PBM on KOA animals, but there is a lack of research comparing different parameters, and the findings remain inconsistent. For instance, Pu Wang et al. demonstrated that 830 nm PBM at 1.5 J/cm^2^ significantly improved 14% of weight distribution on the operated hind paw in anterior cruciate ligament transection (ACLT)-induced KOA rabbits compared to the ACLT mice without laser therapy after 6 and 8 weeks of laser-PBM treatment [43]. This aligns with our findings, where a significant 31% improvement in weight distribution was observed from the sixth week of 940 nm LED therapy. The differences in light sources—laser versus LED—may account for variations in energy delivery efficiency, with LED having lower light coherence potentially requiring higher energy densities to achieve similar effects. Similarly, Eloá F. Yamada et al. showed that 904 nm laser at 18 J/cm^2^ significantly inhibited mechanical hyperalgesia and suppressed spontaneous pain in monosodium iodoacetate (MIA)-induced KOA, which is a rapidly progressing inflammatory KOA model [44]. Gustavo Balbinot et al. reported that 850 nm laser therapy significantly improved weight-bearing and withdrawal thresholds in MIA-induced KOA rats, with an earlier onset of analgesic effects compared to our study [45]. The earlier pain relief in their models may be due to the more acute onset of pain in MIA-induced KOA compared to our DMM model.

A recent review encompassing three in vitro and thirty in vivo studies revealed a significant effect of PBM on cartilage regeneration, with the majority of these studies employing laser technology [16]. The wavelength utilized across these studies ranged from 630 to 904 nm, with energy density ranging from 2 to 1500 J/cm^2^. However, there are limited studies that have explored the use of LED therapy. One study used LED therapy on a KOA model for targeting cartilage [28]. In this study, LED treatment had an up-regulation in the mRNA expressions of type II collagen within the cartilage observed, notwithstanding a lack of significant impact on other cartilage metabolism molecules potentially due to suboptimal treatment parameters. This study utilized two sets of LEDs, possessing wavelengths of 630 nm (red) and 870 nm (infrared), delivering energy amounts of 2 J/cm^2^ (red) and 2.5 J/cm^2^ (infrared) respectively. Additionally, one in vitro study found that a 910 nm laser administering 4 J/cm^2^ and 8 J/cm^2^ irradiation attenuated the IL-1β-induced expression of inflammatory cytokines and matrix metalloproteinases in Chondrocytes [46]. Notwithstanding, this did not extend to a significant impact on *mmp13*, and the study did not evaluate the expression of the extracellular matrix, a crucial indicator for appraising the metabolic state of chondrocytes [46]. Another in vitro study deploying 808 nm laser therapy at 50 J/cm^2^ on chondrocytes uncovered that the latter significantly promoted cellular proliferation, similar to the optimal energy density we found in chondrocytes, 52 J/cm^2^. As far as we can find, there is only one study that evaluated the impact of LED therapy on pain behaviors in animal models of KOA and reported no improvement in pain behavior tests based on gait analysis [27].

Our study stands out by demonstrating the effectiveness of 940 nm LED therapy at 52 J/cm^2^ in reducing spontaneous pain and inhibiting ECM degradation in KOA mice. In contrast to Trevisan et al. who found no improvement in pain behavior with LED therapy at 12 J/cm^2^, our use of a higher energy density and longer wavelength likely enhanced tissue penetration and triggered more significant biological effects. Our cellular studies indicated that lower energy densities (e.g., 13 J/cm^2^) were insufficient to suppress chondrocyte degeneration, underscoring the importance of applying appropriate energy settings. Additionally, the use of different animal models may contribute to variations in findings. While Trevisan et al. used the ACLT model with more pronounced behavioral changes and quicker OA progression [47], our DMM model resulted in a more gradual onset of pain, possibly affecting the therapeutic outcomes. Our research demonstrated a significant reduction in spontaneous pain behavior following therapy with 940 nm wavelength LED at an energy density of 52 J/cm^2^. The different findings between these studies could be related to several key differences in the LED parameters. Our application of a longer wavelength LED (940 nm) may enhance tissue penetration or trigger distinct biological effects, potentially influencing the therapy effectiveness [10]. Additionally, whereas Trevisan et al. employed an energy density of 12 J/cm^2^, our protocol used 52 J/cm^2^ which is from our cell study of chondrocytes. In this study, we applied the same LED parameters from our in vitro experiments to the DMM mouse model, following the direct translation approach previously reported [24]. Our experiments also demonstrated significant protective effects on cartilage and analgesic outcomes in vivo. However, whether more precise strategies for parameter conversion from cell to animal could achieve better effects remains uncertain. Considering the additional complexities in vivo, including skin thickness, tissue composition, and the depth of the target cartilage, future work should aim to establish methods for quantifying and optimizing tissue-specific light delivery in small animals to enhance translational relevance. It is worth noting the potential relevance of a biphasic dose–response relationship with energy density in this therapeutic context [18, 48]. Our results underline the necessity of fine-tuning LED therapy settings to target specified therapeutic goals and suggest that employing a 940 nm wavelength with an energy density of 52 J/cm^2^ may improve spontaneous pain in cartilage-driven KOA.

Numerous molecules have been proposed as the primary photoreceptors or chromophores. The first law of photobiology dictates that for any biological effect to occur, photons must be absorbed by a specific chromophore within cells or tissues [49]. The primary chromophore often discussed in PBM is cytochrome c oxidase (CCO), part of the mitochondrial respiratory chain unit IV [50]. CCO has two absorption bands, one in the red spectral region (600 nm—630 nm) and another in the near-infrared (NIR) spectrum (700 nm ~ 900 nm), aligning with the wavelengths most commonly used in PBM [49]. However, the absorption bands of CCO become much weaker at wavelengths greater than 900 nm [51]. Despite this, beneficial effects of PBM over 900 nm have been observed in nerve regeneration studies, suggesting alternative mechanisms. Recent findings point to the involvement of light-sensitive ion channels, particularly calcium-permeable transient receptor potential (TRP) channels, as key mediators at wavelengths greater than 900 nm [51]. In our study, we observed that both 810 nm and 940 nm LEDs reduced cartilage catabolism, but only 940 nm also enhanced anabolic gene expression. This difference may arise from (1) better tissue penetration of 940 nm light, (2) engagement of alternative photoreceptors beyond CCO, and (3) activation of ion channel–mediated signalling pathways, such as those involving TRP channels and reactive oxygen species (ROS) [52]. While 810 nm is thought to interact primarily with CCO, its efficacy may be limited by shallower tissue penetration, reducing its therapeutic effect in cartilage repair and pain relief.

The distinct effects of wavelengths on ECM degradation may be due to their varying capacities to regulate cartilage catabolism and anabolism. While both 940 nm at 52 J/cm^2^ and 810 nm at 39 J/cm^2^ LED reduced *mmp3* and *mmp13* levels, only 940 nm at 52 J/cm^2^ also stimulated anabolic pathways, increasing *col2a1* and *aggrecan* expression. This suggests that 940 nm is more effective in promoting cartilage synthesis alongside inhibiting degradation. In contrast, 625 nm and 1050 nm LEDs showed no significant protective effects on ECM degradation, likely because these wavelengths do not effectively target photoreceptors for chondrocytes, making 625 nm and 1050 nm less effective in activating the cellular pathways required for regulating cartilage anabolism and catabolism. Although 1050 nm falls within the previously reported 620–1100 nm window that has been shown to elicit biological responses in cells and tissues [53], it did not show protective effects on chondrocytes. A possible explanation is that absorption efficiency of cytochrome c oxidase declines markedly beyond 980 nm, which may limit the biological activity of irradiation at 1050 nm [54]. In addition, 1050 nm coincides with a region of strong water absorption [54]. These factors together may explain the absence of beneficial effects observed with 1050 nm in vitro. Based on these findings in animals, the possible mechanisms and reasons for the differential effects observed for the 940 nm and 810 nm wavelengths of LED in pain relief and cartilage regeneration could be attributed to several factors. Firstly, the more modest effects of 810 nm may relate to its primary interaction with cytochrome c oxidase (CCO), which plays a known role in inflammatory regulation but may not engage anabolic pathways to the same extent. [16, 17, 55]. The therapeutic effects noted with the 940 nm wavelength might be attributed to the interaction with alternate biological targets or mechanisms beyond cytochrome c oxidase, including pathways that regulate reactive oxygen species (ROS) crucial or light-gated ion channel, which has been reported could regulate cartilage regeneration [51, 56–58]. Furthermore, the notion that wavelengths around 940 nm may have a greater skin penetration abilities compared to those near the 810 nm spectrum provides a possible explanation for the noted improvements in cartilage regeneration and pain relief [59]. In our in vitro experiments, non-contact LED irradiation induced only minimal temperature increases in the culture medium: 1.3 °C for 625 nm, 0.2 °C for 810 nm, 0.3 °C for 940 nm, and 0.2 °C for 1050 nm, all measured at room temperature (data not shown). These negligible changes indicate that the observed cellular responses were primarily attributable to PBM rather than thermal effects. Temperature changes were not assessed in vivo, which could be addressed in future studies.

There are some limitations of this study. First, our study only investigated the effects of LED at four specific wavelengths, thus the impact of LED with higher or lower wavelengths, or those that fall in-between, on OA remains unclear. Second, our study was confined to the effects of LED treatment, with the underlying mechanisms not examined; future research is required for more investigation. Third, although the energy density used was based on our previous cellular-level experiments, only a part of the energy could reach the joint cavity in animal experiments. Also, since the period of cell experiments differs from that of animal experiments, and multiple irradiations in the animal knees may cause cumulative effects, we are currently unclear about what the optimal irradiation period and energy setting are in animal experiments. These factors may affect the efficiency of the 940 nm LED therapy. Fourth, sham controls were fully shielded with aluminum foil, unlike LED groups exposed to ambient light. Using non-LED light as a control in future studies could provide a more rigorous comparison. Fifth, this study did not assess additional cellular outcomes such as proliferation and viability. Evaluating these parameters in future work could provide further insight into the effects of PBM on chondrocyte growth and function. Finally, future research needs to confirm the effectiveness of LED therapy in human studies, to verify the safety, efficacy, and optimal treatment protocols of this treatment method in clinical practice.

## Conclusions

In summary, this study demonstrated that 940 nm LED at 52 J/cm^2^ exerted the strongest protective effects on chondrocytes by inhibiting ECM degradation and enhancing anabolic activity. In vivo, 940 nm LED therapy significantly alleviated spontaneous pain and attenuated cartilage degeneration in DMM mice. These findings help define effective LED-PBM parameters for managing knee osteoarthritis by targeting both pain and cartilage degradation.

## Supplementary Information

Below is the link to the electronic supplementary material.

Supplementary Material 1

## Data Availability

The data supporting the findings are presented in the article or further requested from the corresponding author under reasonable necessity.

## Abbreviations

OA: Osteoarthritis
ECM: Extracellular matrix
MMPs: Matrix metalloproteinases
DMOADs: Disease-modifying osteoarthritis drugs
PBM: Photobiomodulation
LED: Light-emitting diode
DMM: Surgical destabilization of the medial meniscus
CCO: Cytochrome c oxidase
NIR: Near-infrared
TRP: Transient receptor potential
ROS: Reactive oxygen species
