# Supplementary material for: Optimizing LED photobiomodulation parameters to prevent cartilage matrix degradation in knee osteoarthritis: in vitro and in vivo study
Source: J Orthop Surg Res. 2025 Oct 29;20:933. doi: 10.1186/s13018-025-06341-7 (PMC12570694; doi:10.1186/s13018-025-06341-7)
Supplement: Supplementary file 1 — Supplementary Material 1 [file 13018_2025_6341_MOESM1_ESM.docx]

Supplementary Materials

Supplementary Table 1 List of primers used in Real-Time PCR.

| **Primer name** | **Forward primer sequence (5’-3’)** | **Reserve primer sequence (5’-3’)** |
| --- | --- | --- |
| GAPDH | GGCAAGTTCAACGGCACAG | CGCCAGTAGACTCCACGAC |
| mmp3 | CTGGAATGGTCTTGGCTCAT | CTGACTGCATCGAAGGACAA |
| mmp13 | CTGCGGTTCACTTTGAGGA | TCTTCTATGAGGCGGGGATA |
| col2a1 | CTCAAGTCGCTGAACAACCA | GTCTCCGCTCTTCCACTCTG |
| aggrecan (ACAN) | AAGTGCTATGCTGGCTGGTT | GGTCTGGTTGGGGTAGAGGT |

Supplementary Table 2 LED therapy parameters for cells

| Peak wavelength (nm) | 625, 810, 940 or1050 | | | | | | | |
| --- | --- | --- | --- | --- | --- | --- | --- | --- |
| Power density (mW/cm^2^) | 44 | | | | | | | |
| Spot size (cm^2^) | 3.4 | | | | | | | |
| Frequency | Continuous output | | | | | | | |
| Distance from the cultures (mm) | 12 cm | | | | | | | |
| Groups | Non-radiated | TNF-α + Non-radiated | TNF-α + LED | TNF-α + LED | TNF-α + LED | TNF-α + LED | TNF-α + LED | TNF-α + LED |
| Irriadiation time (min) | 0 | 0 | 5 | 10 | 15 | 20 | 25 | 30 |
| Energy density | 0 | 0 | 13 | 26 | 39 | 52 | 65 | 78 |
